# Protective effects of *Cassia tora* leaves in experimental cataract by modulating intracellular communication, membrane co-transporters, energy metabolism and the ubiquitin-proteasome pathway

**DOI:** 10.1080/13880209.2017.1299769

**Published:** 2017-03-08

**Authors:** V. Sreelakshmi, Annie Abraham

**Affiliations:** Department of Biochemistry, University of Kerala, Thiruvananthapuram, Kerala, India

**Keywords:** Blindness, functional food, selenite cataract, oxidative stress

## Abstract

**Context:** Cataract is the clouding of eye lens which causes impairment in vision and accounts for the leading factor of global blindness. Functional food-based prevention of cataract finds application in vision research because of its availability and easy access to all classes of the society. *Cassia tora* Linn. (Caesalpinaceae) is an edible plant mentioned in the traditional systems of medicine for whole body health, especially to the eyes.

**Objective:** The present study evaluates the potential of ethyl acetate fraction of *Cassia tora* leaves (ECT) on experimental cataract.

**Materials and methods:** Cataract was induced by a single subcutaneous injection of sodium selenite (4 μg/g body weight) on 10th day. ECT was supplemented orally from 8th day up to 12th day at a concentration of 5 μg/g body weight and marker parameters were evaluated after 30 days.

**Results:** The production of MPO and the activation of calpain were reduced 52.17% and 36.67% by ECT in lens tissue, respectively. It modulated the energy status by significantly increasing the activity of CCO 1 (55.56%) and ATP production (41.88%). ECT maintained the ionic balance in the lens by reducing the level of sodium (50%) and increasing the level of potassium (42.5%). It also reduced cell junction modifications and preserved a functional ubiquitin-proteasome pathway.

**Discussion and conclusion:** The results reinforce the growing attention on wild plant food resources for preventive protection against cataract. The data suggest the value of *Cassia tora* leaves as a functional food for ameliorating cataract pathology.

## Introduction

Visual impairment is any visual condition that affects an individual’s ability to successfully complete the day to day activities of life. Uncorrected refractive errors, cataract, glaucoma, etc., are the major causative factors of visual impairment. Cataract is the clouding of the lens, which initially reduces the clarity of the vision and eventually progresses to blindness if left untreated. According to the World Health Organization (WHO) estimates of 2010, 51% of global blindness is due to cataract (Pascolini & Mariotti 2010).

The eye lens is a transparent organelle devoid of blood supply and the lens proteins do not undergo any turnover during one’s whole life. It is a unique tissue by its architecture and any factor that affects the normal physiology of lens forms opacity and cataract. The major factors responsible for cataract formation are believed to be the oxidative attack to lens proteins, lipids and lenticular epithelial cells, calcium accumulation (Gao et al. 2004), lens epithelial cell apoptosis (Lee et al. 2002), protein aggregation (Moreau & King 2012), inflammatory responses (Uchio et al. 1998), lenticular cell junction modification (Berthoud & Beyer 2009), disturbance in energy production (Balog et al. 2001), disturbed ubiquitin-proteasome pathway (Viteri et al. 2004), etc. In spite of the availability of intraocular lens implants, cataract remains the leading cause of blindness in developing countries. In addition to this, many post-operative complications such as endo-epithelial corneal oedema, cystoid macular oedema, uveitis, haemorrhagic complications, bullous keratopathy, infection, double vision, high or low eye pressure, secondary cataract, etc. were reported (Koos et al. 2003).

Phytomedicine is of great significance in the present system of medicine and renewed attention has been set to find out the relationship between dietary nutrients and disease prevention. Nutraceuticals are naturally derived bioactive substances present in foods, dietary supplements and herbal products that have health-boosting and disease preventing properties (Pandey et al. 2011). Functional foods are a class of nutraceutical that improves health through daily diet and has gained much attention due to its cost effective and easily accessible characteristics. In the field of vision research, a large number of studies are focusing on the importance of exploring the prospect of natural resources to delay the onset and progression of cataract. Many medicinal plants and their formulations are reported to offer preventive protection against cataract (Lija et al. 2006; Rooban et al. 2009; Sasikala et al. 2010).

*Cassia tora* Linn. (Caesalpinaceae) is an edible, annual herb growing as a weed. The young leaves and immature pods of *Cassia tora* are consumed as a vegetable and its roasted mature seeds are widely used as a healthy beverage (Cherng et al. 2008). The leaves, flowers, fruits, seeds and roots are reputed for their medicinal properties. According to Ayurveda, the leaves and seeds are ophthalmic, liver tonic, cardiotonic and antimicrobial and scientifically validated for pharmacological activities such as hypolipidemic, hypoglycemic, anticancer etc. (Arulpandi & Kanimozhi 2011). HPLC analysis of ECT indicates the presence of kaemferol, chrysophanol and emodin when compared to the retention time of corresponding standards. Besides the previous compounds, ESI-MS of ECT revealed one more anthraquinones physcion. The identification of compounds by ESI-MS is based on the comparison with standards from a library of plant phenolics (Figures S1 and S2) (Sreelakshmi & Abraham 2016b). The present work is an extension of our previous studies on the attenuation of cataract pathology by reducing protein and lipid modifications by *Cassia tora* leaves on oxidative stress cataract models (Sreelakshmi & Abraham 2016a, 2016b, 2016c). The current study is designed to evaluate the mechanism of anti-cataractogenic effect of edible *Cassia tora* leaves on selenite-induced animal models by focusing on membrane integrity, energy metabolism, proteolysis and ubiquitin-proteasome pathway in the lens.

## Materials and methods

### Chemicals

All the chemicals and biochemicals used were of analytical grade and purchased from Sigma India, SRL, Ranbaxy and Spectrochem, India.

### Plant material

*Cassia tora* leaves were collected from Kariavattom campus in June–August 2014, authenticated by Dr. G. Valsala Devi, Curator, Department of Botany, University of Kerala and a voucher specimen was deposited there (Accession No: KUBH 5844). The leaves were shade dried and extracted with 80% methanol, filtered and the solvent was evaporated in vacuum at 40 °C on a rotary evaporator (Heidolph LABOROTA 4000 efficient). The crude extract was partitioned successively using petroleum ether, ethyl acetate, butanol and water. Each fraction was concentrated again and the bulk of the antioxidant activity was showed by ethyl acetate fraction of *Cassia tora* leaves (ECT). This fraction was dissolved in PBS (prepared in sterile water) for the animal experiments.

### Animals

Sprague–Dawley rat pups at 8–10 days postpartum were housed along with their mother in polypropylene cages under a day/night cycle of 12 h, at 25 ± 1 °C room temperature. The rats received laboratory chow (Hindustan Lever Ltd., India) and distilled water. All the ethical guidelines were followed for the conduct of animal experiments in strict compliance with institutional animal ethical committee (IAEC) and committee for the purpose of control and supervision of experiments on animals (CPCSEA), Government of India (IAEC-KU-5/2012-13, BC. AA32b).

### Experimental procedures

A dose response study was carried using ECT at different concentrations (1, 2.5, 5 and 10 μg/g body weight) against selenite-induced cataract. The rat pups were grouped into four with six pups in each group.

**Group I**       Normal

**Group II**    Selenite 4 μg/g body weight

**Group III** Selenite 4 μg/g body weight + ECT 1 μg/g body weight

**Group IV** Selenite 4 μg/g body weight + ECT 2.5 μg/g body weight

**Group V** Selenite 4 μg/g body weight + ECT 5 μg/g body weight

**Group VI** Selenite 4 μg/g body weight + ECT 10 μg/g body weight

Group II–VI were given a single subcutaneous injection of sodium selenite (4 μg/g body weight) on the 10th day (Ostadalova et al. 1978) while ECT was administered by gastric intubation from the 8th day up to 12th day at different concentrations to group III and VI. Cataract could be visualized from the 15th day with the help of an ophthalmoscope and later on with the naked eye. The animals were euthanized by sodium pentothal injection on the 30th day and the lens was extracted through posterior approach. The minimal effective dose was fixed as 5 μg/g body weight by measuring the activities of superoxide dismutase and catalase. The rat pups were grouped into four with six in each group.

**Group I**  Normal

**Group II**  Normal + ECT (5 μg/g body weight)

**Group III**  Sodium selenite (4 μg/g body weight)

**Group IV** Sodium selenite (4 μg/body weight) + ECT (5 μg/g body weight)

Group III and group IV were given a single subcutaneous injection of sodium selenite (4 μg/g body weight) on the 10th day (Ostadalova et al. 1978) while ECT was administered by gastric intubation from the 8th day up to 12th day at a concentration 5 μg/g body weight to group II and IV.

### Analytical procedures

Activity of superoxide dismutase was measured by the method of Kakkar et al. (1984), catalase was measured by the method of Aebi (1984), myeloperoxidase (MPO) was assayed by the method described by Desser et al. (1972), calpain was assayed by the method of Ross and Schatz (1973). Cytochrome C oxidase 1 (CCO 1) activity was assayed by ELISA in the mitochondrial fraction obtained as per the procedure of kit procured from BioVision, USA. ATP was assayed spectrophotometrically by the method of Williamson and Corkey (1969). Protein values by the method of Lowry et al. (1951).

### Analysis of sodium and potassium in the lens

The lenses were weighed, cleaned and washed with phosphate buffered saline. Then the tissue was digested with the necessary volume of concentrated nitric acid: perchloric acid mixture (5:1). After complete digestion, the lenses were dried and diluted with 1% nitric acid. The analysis was carried by atomic absorption spectroscopy (Shimadzu) operated with a slit width of 589.6 nm for sodium, 766.5 nm for potassium using specific standards.

### Isolation of RNA from lens and RT-PCR study

RNA was isolated from rat lens using trizol reagent (Sigma-Aldrich, St. Louis, MO) as described by Chomczynski and Sacchi (1987). The RNA concentration was determined from the absorbance at 260 nm (Bio Photometer, Eppendorf AG, Hamburg, Germany). All samples had a 260/280 nm absorbance ratio 1.78 ± 0.06. RT-PCR and PCR amplifications were carried out using kits from Thermo Scientific, India. The primer sequences used are listed in Table 1. Initial PCR activation for 15 min at 95 °C was followed by 3 steps of cycling process. Each cycle consists of denaturation for 1 min at 94 °C, annealing for 1 min at 65 °C, extension for 1 min at 72 °C, repeated for 37 cycles and final extension for 10 min at 72 °C. The PCR products were electrophoresed on 1% agarose gels containing 0.05 μg/mL ethidium bromide. The mRNA expression was quantified using a phosphor-imager and using the image quant software and the relative expression was compared and normalized to the expression of β actin in the same sample.

**Table 1.** Rat-specific PCR primer sequences.

Genes	Forward primer	Reverse primer
Calpain 2	5′ TGTGGACAACCCAGTTACAAGC3′	5′ AGGATTCCATCTCAACACGACA3′
MIP	5′CACCAGCTGTCCGAGGAAA3′	5′GCGTCAGGAAGATCTCCACAGT3′
CCO 1	5′ TCACAGTAGGGGGCCTAACA3′	5′GGCTTTTGCTCATGTGTCATT3′
E Cadherin	5′GACATCATCACTGTGGCAGC3′	5′TCTGCAGCAACAGTGTGGAC3′
N Cadherin	5′ACACTCAAGGTGACTGA3′	5′CGCGCAGTGTAAGATGCGAT3′
NKCC1	5′GTCACATACACTGCCGAAAG3′	5′TCTGCGATTCCAACAACATA3′
ube1	5′TCATGGAGCGGACACTG3′	5′CCTTCTCGAAATCAATGGG3′
ubc2 (E2)	5′AGAATCCACAAGGAATTGAATGA3′	5′TACATGGCATACTTCTGAGTCCA3′
β actin	5′TCCTGTGGCATCCATGAAACTAC3′	5′AGCACTGTGTTGGCATAGAGGTC3′

### Protein expression analysis

Indirect ELISA was performed according to the method of de Echaide et al. (2005) using specific antibodies.

### Statistical analysis

All statistical calculations were carried out (Steel et al. 1996) with the package for social sciences (SPSS) software program (version 17.0 for Windows). The values were expressed as the mean ± SEM. The data were statistically analyzed using one-way analysis of variance and the significant difference of means was determined using Duncan’s multiple range tests at the level of *p* < 0.05. Comparison is carried out between the groups; cataract group was compared with normal group and treatment group was compared with cataract group.

## Results

### Dose response study

**Figure 1.**
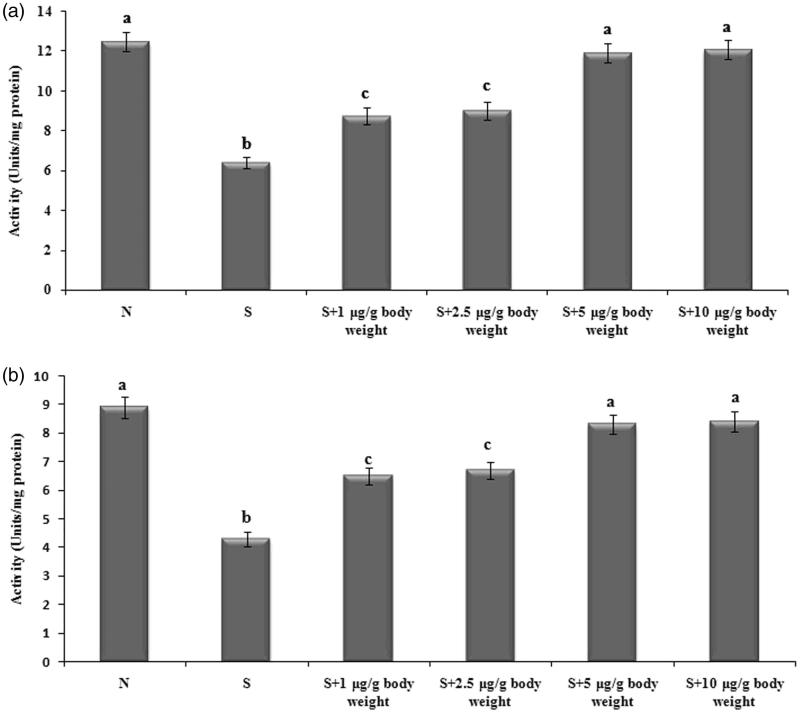
Activity of superoxide dismutase (a) and catalase (b) in different experimental groups. Each value represents mean ± SEM of six values. N: Normal; S: Selenite. Different alphabets indicate significant difference between different groups at *p* < 0.05. Comparison is carried out between the groups; Cataract group is compared with control group and treatment group is compared with cataract group. ^a^indicates normal group, ^b^indicates significantly different from normal group and ^c^indicates significantly different from cataract group.

The activities of antioxidant enzymes superoxide dismutase and catalase were significantly lowered in the selenite-induced group in comparison with the lenses of normal rats. But the activities were restored by ECT pretreatment and were normalized significantly by ECT at the doses 5 and 10 μg/g body weight when compared with cataractous lenses. The least effective dose 5 μg/g body weight was selected for further analysis (Figure 1). ECT pretreatment at the dose of 5 μg/g body weight resulted in 1.85 and 1.94 fold increase in the activity of superoxide dismutase and catalase, respectively.

### Activity of MPO

**Figure 2.**
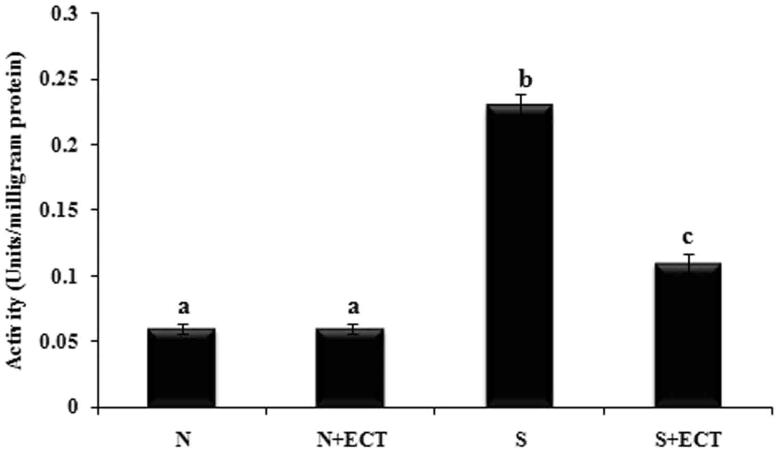
Activity of MPO in the lenses of experimental groups. N: Normal; S: Selenite. Each value represents mean ± SEM of six values. Different alphabets indicate significant difference between different groups at *p* < 0.05. Comparison is carried out between the groups; ^a^indicates normal group, ^b^indicates significantly different from normal group and ^c^indicates significantly different from cataract group.

The activity of pro-inflammatory enzyme MPO was increased in the lenses of selenite-induced animals compared to normal ones. But the administration of ECT significantly reduced its activity in the group IV animals (Figure 2).

### Proteolysis in the lens

**Figure 3.**
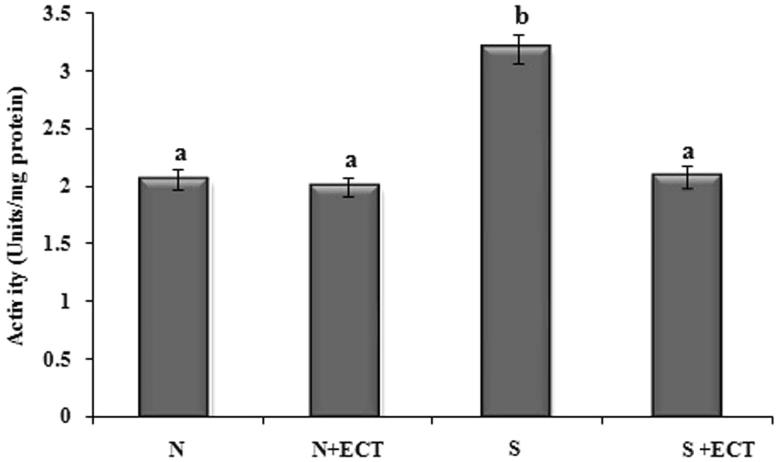
Activity of calpain in the lenses of experimental groups. N: Normal; S: Selenite. Each value represents mean ± SEM of six values. Different alphabets indicate significant difference between different groups at *p* < 0.05. Comparison is carried out between the groups; ^a^indicates normal group, ^b^indicates significantly different from normal group and ^c^indicates significantly different from cataract group.

**Figure 4.**
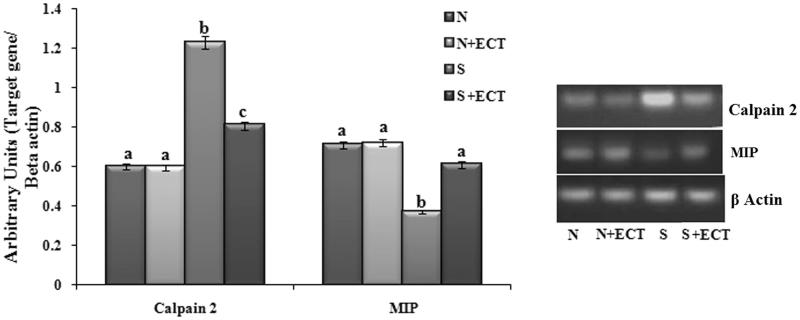
Photographic and graphical representation of mRNA expression of calpain and MIP in the lenses of different experimental groups. N: Normal; S: Selenite. Each value represents mean ± SEM of six values. Different alphabets indicate significant difference between different groups at *p* < 0.05. Comparison is carried out between the groups; Cataract group is compared with control group and treatment group is compared with cataract group. ^a^indicates normal group, ^b^indicates significantly different from normal group and ^c^indicates significantly different from cataract group.

The activity and gene expression of calpain were raised in the lenses of group III animals. However, the pretreatment of ECT in group IV resulted in a marked decrease (1.51-fold) in the level and expression of calpain (Figures 3 and 4).

### Cellular communication in the lens

**Figure 5.**
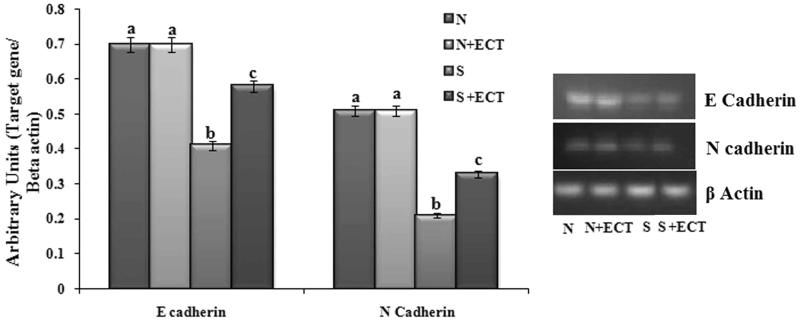
Photographic and graphical representation of mRNA expression of E Cadherin and N Cadherin in different experimental groups. N: Normal; S: Selenite. Each value represents mean ± SEM of six values. Different alphabets indicate significant difference between different groups at *p* < 0.05. Comparison is carried out between the groups; Cataract group is compared with control group and treatment group is compared with cataract group. ^a^indicates normal group, ^b^indicates significantly different from normal group and ^c^indicates significantly different from cataract group.

Gene expression of major intrinsic protein (MIP) and cadherins (E cadherin and N cadherin) were considerably altered in the selenite-induced group. The expressions were increased (MIP: 1.65-fold, E cadherin: 1.41-fold and N cadherin: 1.57-fold) by ECT supplementation (Figures 4 and 5).

### The level of ions and ion transporter in the lens

**Figure 6.**
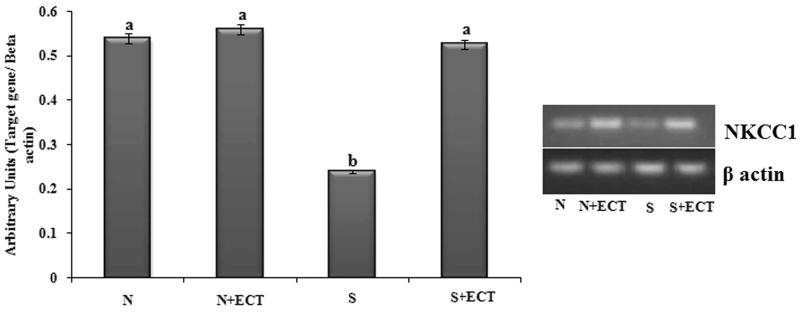
Photographic and graphical representation of mRNA expression of NKCC 1 in different experimental groups. N: Normal; S: Selenite. Each value represents mean ± SEM of six values. Different alphabets indicate significant difference between different groups at *p* < 0.05. Comparison is carried out between the groups; Cataract group is compared with control group and treatment group is compared with cataract group. ^a^indicates normal group, ^b^indicates significantly different from normal group and ^c^indicates significantly different from cataract group.

The level of sodium and potassium were respectively raised and reduced in the lenses of selenite induced animals. Their level was normalized in ECT pretreated lenses (Table 2). Gene expression of sodium potassium chloride co-transporter 1 (NKCC1) was reduced in the cataractous lenses and was enhanced (2.2-fold) by ECT supplementation (Figure 6).

**Table 2.** Level of sodium and potassium in the lenses of different experimental groups.

Groups	Sodium (μg/g lens)	Potassium (μg/g lens)
N	130.71 ± 5.33^a^	896.90 ± 36.58^a^
N + ECT	129.62 ± 5.29^a^	900.7 ± 36.74^a^
S	375.8 ± 15.34^b^	378.6 ± 15.45 ^b^
S + ECT	186.65 ± 7.61^c^	658.47 ± 26.87^c^

N: Normal; S: Selenite. Each value represents mean ± SEM of six values. Different superscript alphabets indicate significant difference between different groups at *p* < 0.05. Comparison is carried out between the groups; Cataract group is compared with control group and treatment group is compared with cataract group. ^a^indicates normal group, ^b^indicates significantly different from normal group and ^c^indicates significantly different from cataract group.

### Energy metabolism in the lens

**Figure 7.**
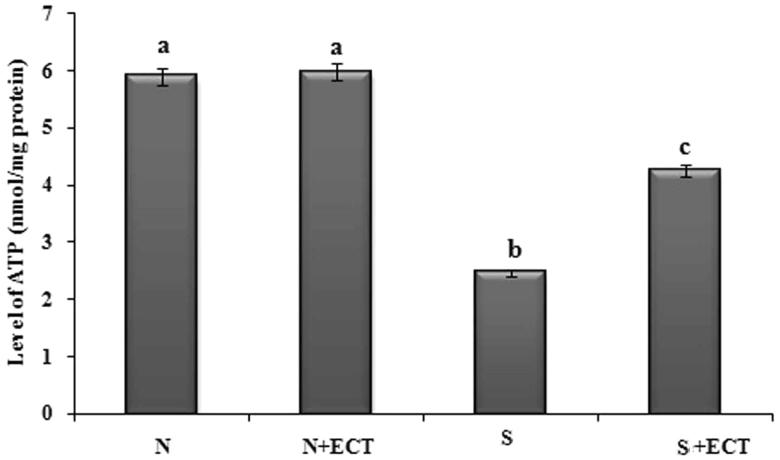
The level of ATP in the lenses of experimental groups. N: Normal; S: Selenite. Each value represents mean ± SEM of six values. Different alphabets indicate significant difference between different groups at *p* < 0.05. Comparison is carried out between the groups; ^a^indicates normal group, ^b^indicates significantly different from normal group and ^c^indicates significantly different from cataract group.

**Figure 8.**
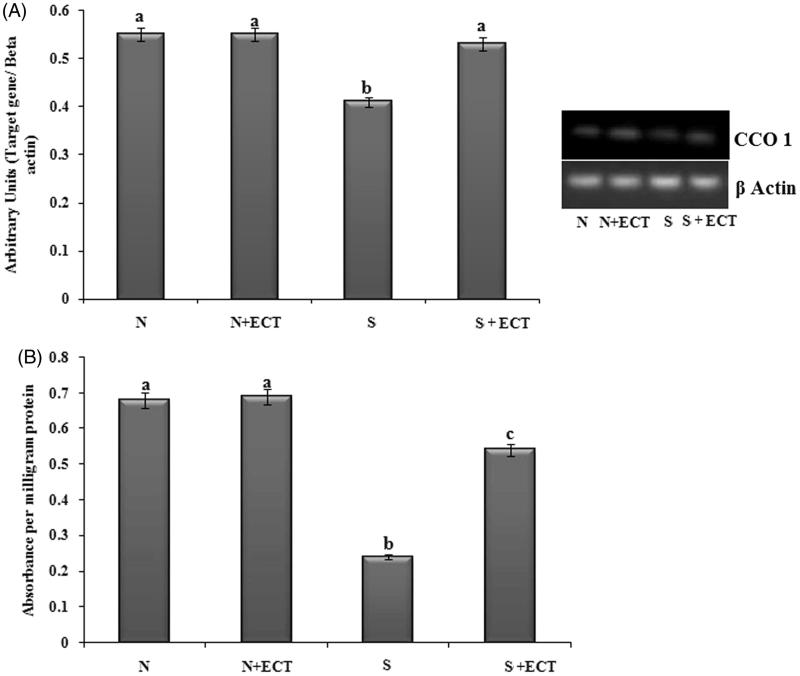
(A) Photographic and graphical representation of mRNA expression of CCO 1 in different experimental groups. (B) Protein expression of CCO 1 in the lenses of different experimental groups. N: Normal; S: Selenite. Each value represents mean ± SEM of six values. Different alphabets indicate significant difference between different groups at *p* < 0.05. Comparison is carried out between the groups; ^a^indicates normal group, ^b^indicates significantly different from normal group and ^c^indicates significantly different from cataract group.

A decrease in the level of ATP was observed upon administration of selenite in group III animals and the level was improved by ECT pretreatment in group IV lenses (Figure 7). The gene transcript of CCO 1 was found to be reduced in the cataract group and ECT supplementation resulted in the 1.29-fold increase in their expression in group IV. Protein expression of cytochrome C also confirmed the anti-cataractogenic potential of ECT (Figure 8(A,B)).

### Ubiquitin-proteasome pathway in the lens

**Figure 9.**
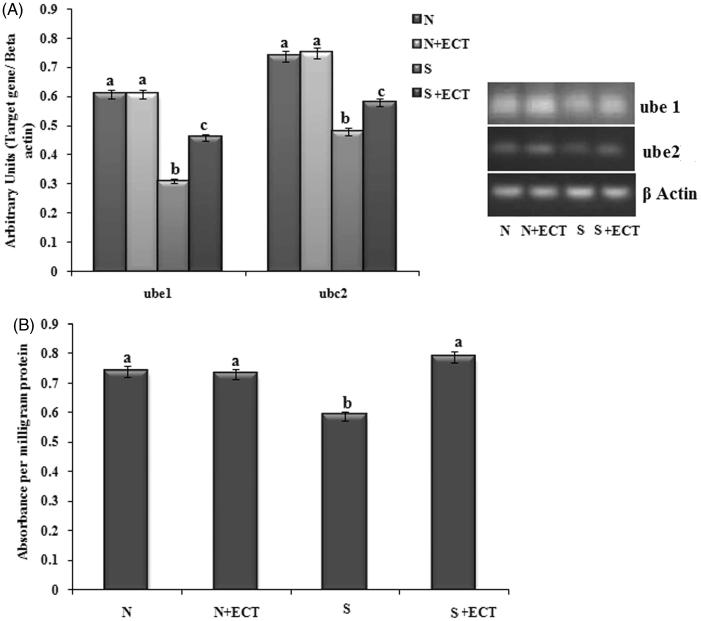
(A) Photographic and graphical representation of mRNA expression of Ub e1 and Ub e2 in different experimental groups. (B) Protein expression of Ub E1 in the lenses of different experimental groups. N: Normal; S: Selenite. Each value represents mean ± SEM of six values. Different alphabets indicate significant difference between different groups at *p* < 0.05. Comparison is carried out between the groups; Cataract group is compared with control group and treatment group is compared with cataract group. ^a^indicates normal group, ^b^indicates significantly different from normal group and ^c^indicates significantly different from cataract group.

To determine the effect of the ECT on ubiquitin-proteasome function, the gene expression of Ube1 and Ube2 were determined. The gene transcript levels of Ube1 and Ube2 and protein level expression of Ube1 were down-regulated in selenite challenged, untreated rat lenses. The expressions were found to be significantly up-regulated (Ube1: 1.48-fold and Ube2: 1.21) in the lenses of ECT pretreated animals (Figure 9(A,B)).

## Discussion

Opacification of eye lens by cataract is the major reason for global blindness and due to its incurable nature, many pharmacological strategies have been proposed for its prevention. Functional food-based herbal medicine is a novel approach for the management of cataract. *Cassia tora* is a nutritious medicinal plant employed widely in the traditional medical system for whole body rejuvenation with specific action on eyes. In this study, the effect of *Cassia tora* leaves was evaluated in the animal model. Selenite-induced cataract model was selected for the study because of its universal acceptance that it possesses all the features of age-related cataract (Shearer et al. 1997). ECT at the doses 5 and 10 μg/g body weight normalized the activities of antioxidant enzymes and the minimal effective dose was fixed as 5 μg/body weight for further studies.

MPO is a pro-oxidative and pro-inflammatory enzyme in the biological system that produces various oxidants such as hypochlorous acid, hydroxyl radicals, singlet oxygen, ozone, and nitrite ion. In the lens tissue, hypochlorite and hydroxyl radicals are associated with modification of lenticular proteins and lipids, protein misfolding, lipid peroxidation, lens epithelial apoptosis, etc., and all these disturb lens membrane permeability (Zhang et al. 2002; Barbusinski 2009; Buschini et al. 2011). Enhanced levels of MPO and its oxidative products were reported in macular degeneration and cataractogenesis (Yugai et al. 1992; Marsili et al. 2004; Michal et al. 2010). Our study is in line with above reports that the activity of MPO was found to be raised in selenite-induced cataractous lenses. ECT pretreatment prevented the upsurge of MPO suggesting the possible role of ECT in reducing the inflammatory response and oxidant signalling associated with cataract.

Calpains are a family of calcium-activated, neutral, non-lysosomal, cysteine proteases. Cells that contain calpain also have its endogenous inhibitor; calpastatin in concentrations sufficient to completely block the over-expression of calpain. The increase in the calcium concentration results in calpain activation which leads to irreversible tissue damage. At the pathological level, over-activation of calpain activity has been implicated in neurodegenerative diseases, cataract formation, etc. (Suzuki & Sorimachi 1998). Calpain-induced proteolysis and precipitation of crystallins constitute an underlying biochemical mechanism for many types of cataracts (Huang & Wang 2001; Ueda et al. 2002). In the present study, both the activity and mRNA expression of calpain 2 were elevated upon cataract induction in group III lenses and was normalized by ECT pretreatment in group IV explaining the potential of ECT in maintaining lens transparency through the prevention of calpain activation.

Major intrinsic proteins (MIP) form aqueous pores and allow passive transport of their solute(s) across the membrane with selectivity (Tamir & Thomas 2006). Growing evidence suggests the abnormal truncation or loss of MIP as a mechanism of cataract formation by alteration of gating of MIP channels and its adhesive characters (Boyle & Takemoto 1999; Grey et al. 2003). Abnormal phosphorylation, deamidation, N and C-terminal truncation and proteolysis of MIP by m-calpain (Schey et al. 2000; Ma et al. 2005) were reported in the aged lenses. In our study, loss of gene transcript of MIP was observed in cataractous lenses as reported by Schey et al. (1999) on the effect of selenium on MIP. The level of MIP was normalized by ECT pretreatment as evidenced by the reduced activity of calpain 2. It is reported that *Cassia tora* leaves have the ability to reduce the enzyme transglutaminase-2, an inducible enzyme that deamidates lens proteins on cataract (Sreelakshmi & Abraham 2016b) and the maintenance of MIP might be through the inhibition of transglutaminase.

Cadherins are a family of calcium dependent-cell adhesion molecules functioning in cell–cell interaction (Perez-Moreno et al. 2003). Proper cell adhesion maintains stable cellular organization and its disruption is associated with cataract formation (More et al. 2001). In the present study, the mRNA level expressions of E cadherin and N cadherin were altered in cataractous lenses and this observation is in line with the report of Lyu et al. (2003) on steroid cataract. The levels of cadherins are normalized by ECT pretreatment. Altered cadherins on selenite models and the possible role of *Cassia tora* leaves administration on modulating the modification of cell adhesion molecules and maintaining lens transparency is reported for the first time from our group.

The changes in the composition of electrolytes such as increase in sodium and decrease in potassium are lethal to the lens and contribute to the failure of transparency by water accumulation, cell swelling and consequent disturbance in protein synthesis (Shinohara & Piatigorsky 1977; Shukla et al. 1996). In addition to a sodium-potassium pump, lens membrane is equipped with NKCC, a class of ion transport proteins that transport Na, K, and Cl ions into and out of cells in an electrically neutral manner, with a stoichiometry of 1Na:1 K:2Cl (Haas & Forbush 2000). Reduced level of NKCC 1 gene transcript in the cataractous lens is reflected in the higher level of sodium and lower level of potassium. ECT administration prevented these changes in the electrolyte levels and maintains lens homeostasis. Quercetin is one of the active principles identified in the *Cassia tora* leaves (Vijayalakshmi & Geetha 2014) and quercetin has shown a similar effect on diabetic cataract (Ramana et al. 2007). The effect of ECT may be attributed to the presence of quercetin in it.

Lens requires a continuous supply of energy and CCO is the terminal enzyme of mitochondrial electron transport chain that plays the major role in the generation of ATP (Lyu et al. 2003). The current observation of reduced gene expression profile of CCO 1 and the activity of CCO in the cataractous lenses is supported by the report of Fariss et al. (2005). They had reported that oxidative damage down regulates the mitochondrial genome and is reflected in the decreased level of ATP in cataract. ECT effectively preserves lens metabolism by maintaining ATP level and CCO expression. Our results are in line with the reports of researchers who have used aminoguanidine and caffeine for preventing cataract pathology (Nagai & Ito 2007; Varma et al. 2010). The results of this study highlighted the ability of *Cassia tora* leaves in alleviating cataract pathology by preventing membrane oxidation and permitting an unbroken delivery of energy to lens epithelial cells.

Oxidant damage to lens proteins and its accumulation, aggregation and precipitation are implicated in the formation of cataract. For maintaining the lens transparent, the damaged proteins should be removed and the process is done by ubiquitin-proteasome mechanism. Ubiquitin-dependent protein degradation process requires the co-ordinated reactions of three enzymes. In the first step, adenylation of ubiquitin molecule is carried out by ubiquitin-activating enzyme (Ub E_1_) by hydrolyzing ATP and adenylated ubiquitin is then transferred to ubiquitin-conjugating enzyme (Ub E_2_/C_2_). In the final step ubiquitin ligases (Ub E_3_) recognizes the specific protein to be ubiquitinated and catalyzes the transfer of ubiquitin from E_2_ to the target protein and target to 26 S proteasome for proteolysis with the recycling of ubiquitin (Ciechanover 1994). Ubiquitin-dependent proteasome pathway is under the control of oxidant response (Shang et al. 1997). In the current study, the activities of both activating and conjugating enzymes were altered in the cataractous lens were normalized by ECT treatment as evidenced by the ELISA results of Ub E1. Reports are available on the role of a defective ubiquitin-proteasome event in the hyper-activation of calpain and cataract formation (Liu et al. 2015). The observed reduction in calpain activation might be the reason for restoration of the functional ubiquitin-proteasome pathway. Our data supporting the effect of ECT in cataract prevention adds a novel practical approach for the management of cataract blindness.

## Conclusions

**Figure 10.**
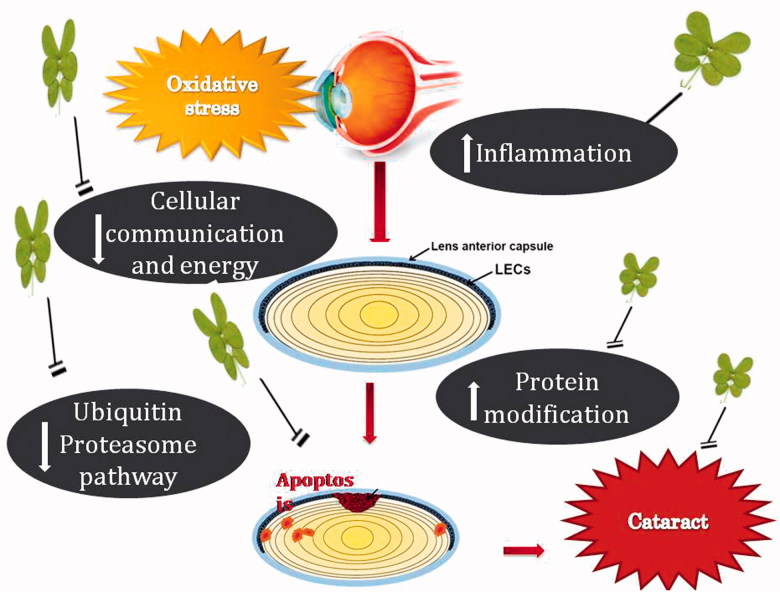
Biochemical events associated with the protective effect of *Cassia tora* in cataract.

This investigation suggests that *Cassia tora* leaves offer-effective protection against cataract by reducing the inflammatory response, suppressing protein denaturation and cross-linking, maintaining membrane integrity and proper intercellular communication with the subsequent maintenance of lens metabolism (Figure 10). On the basis of the above study, it might be concluded that *Cassia tora* leaves possess a significant role in the prevention/management of cataract and supports its traditional usage in the treatment of various eye disorders and for eye rejuvenation. The protective potential of *Cassia tora* leaves might be due to the additive effect of phytochemicals present in the leaves. Incorporation of this functional plant food in the daily diet is a better alternative in the preventive therapy against cataract.

## Supplementary Material

Annie_Abraham_et_al_supplemental_content.zip
