# Supplementary figures and images for: Protective effects of Cassia tora leaves in experimental cataract by modulating intracellular communication, membrane co-transporters, energy metabolism and the ubiquitin-proteasome pathway
Source: Pharm Biol. 2017 Mar 8;55(1):1274–82. doi: 10.1080/13880209.2017.1299769 (PMC6130452; doi:10.1080/13880209.2017.1299769)

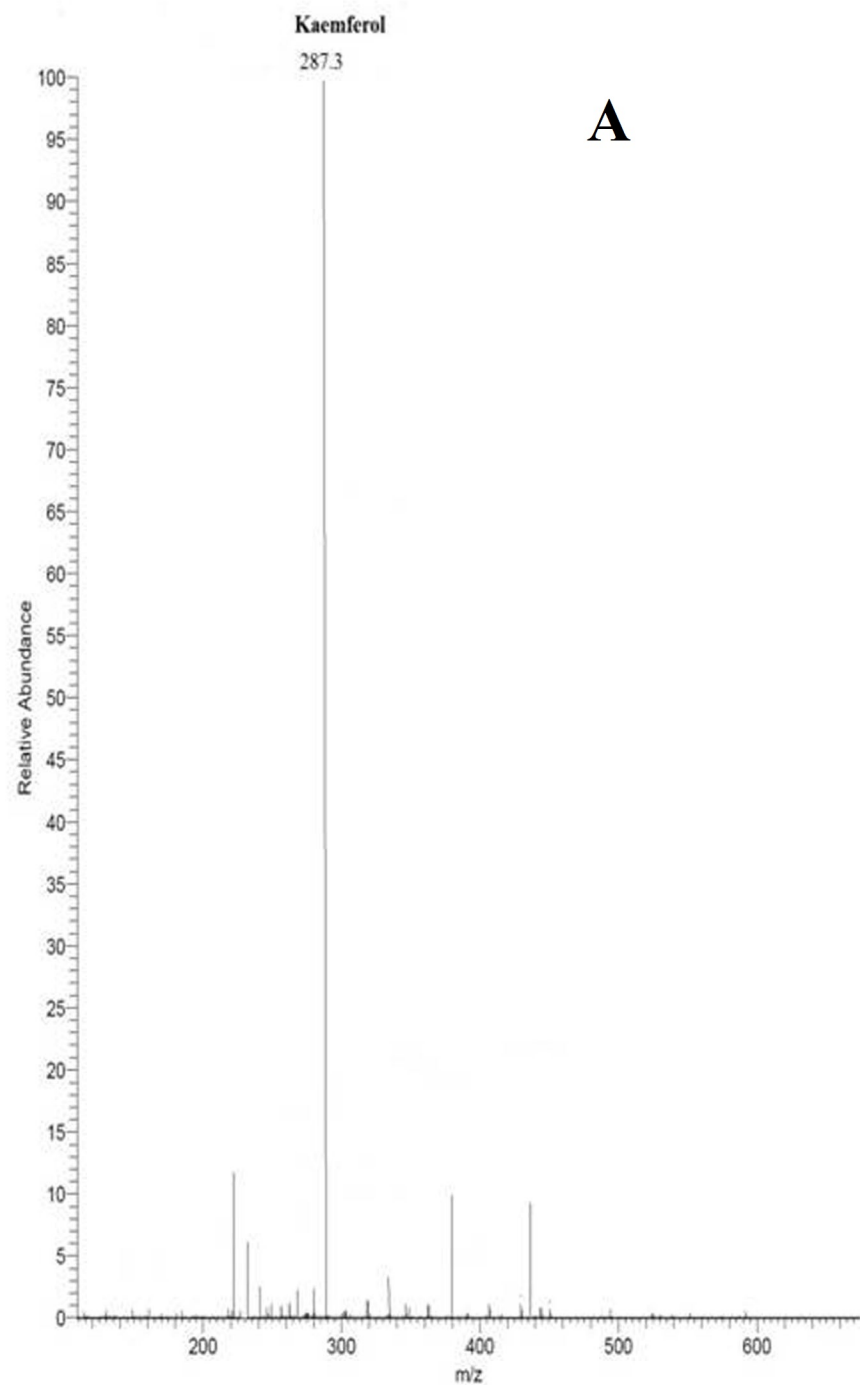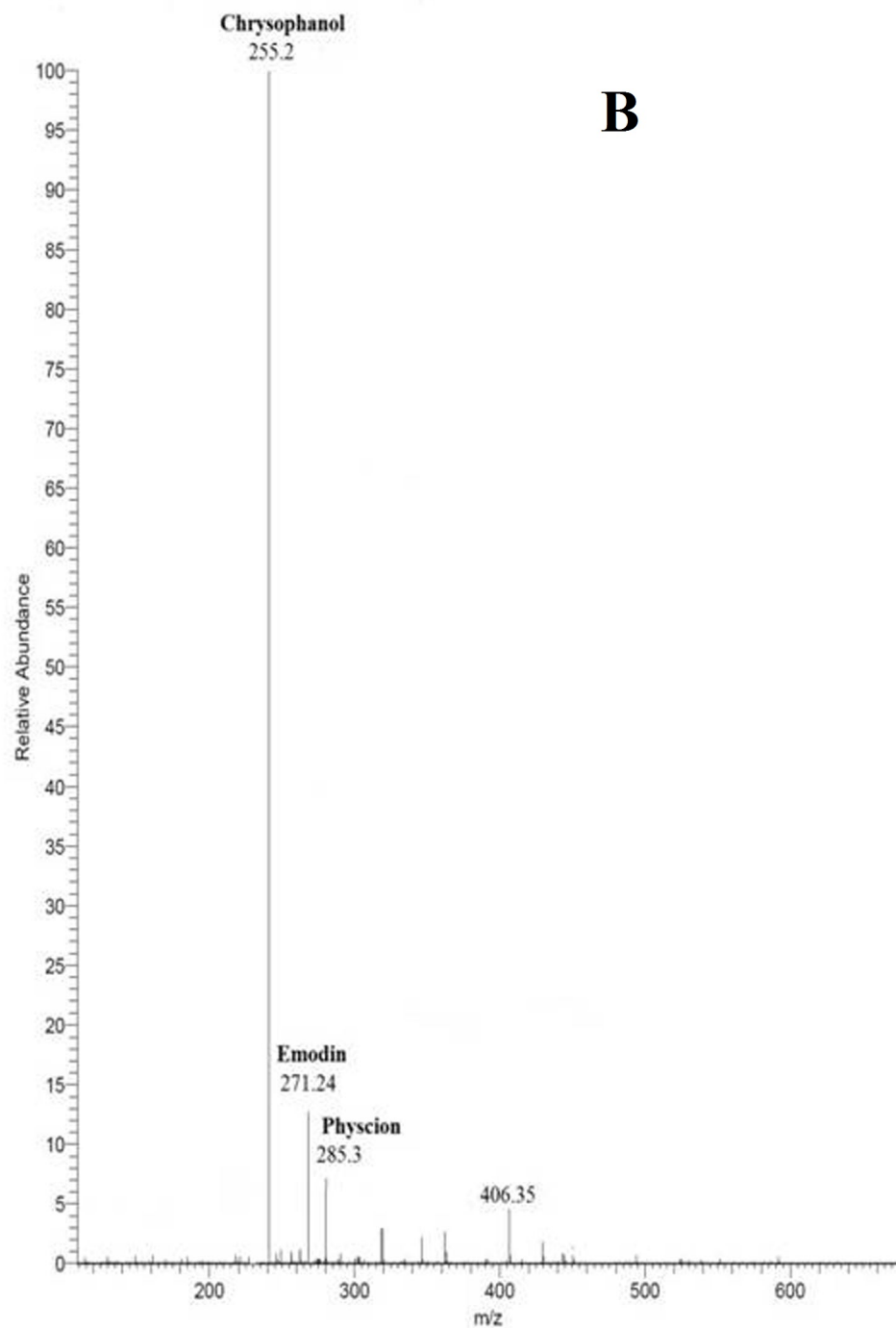

**A**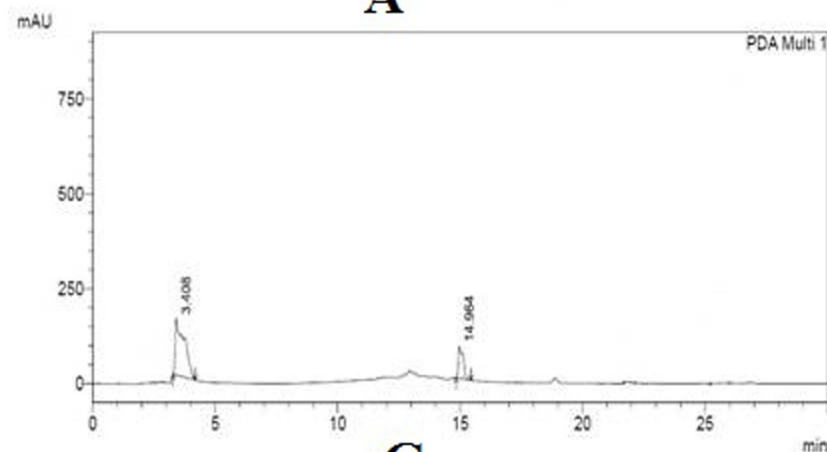**B**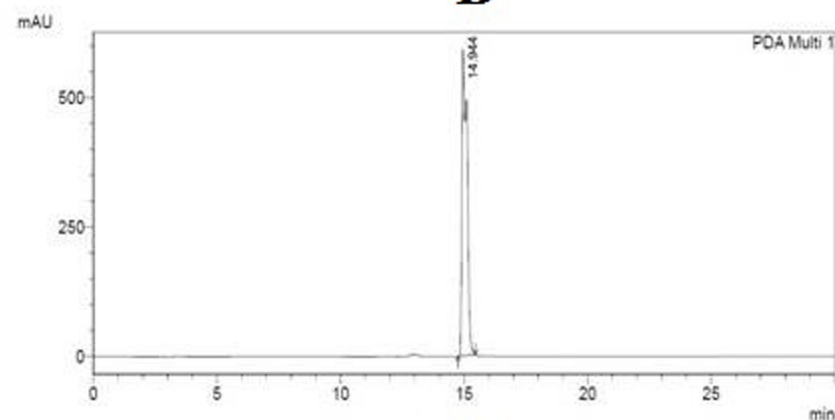**C**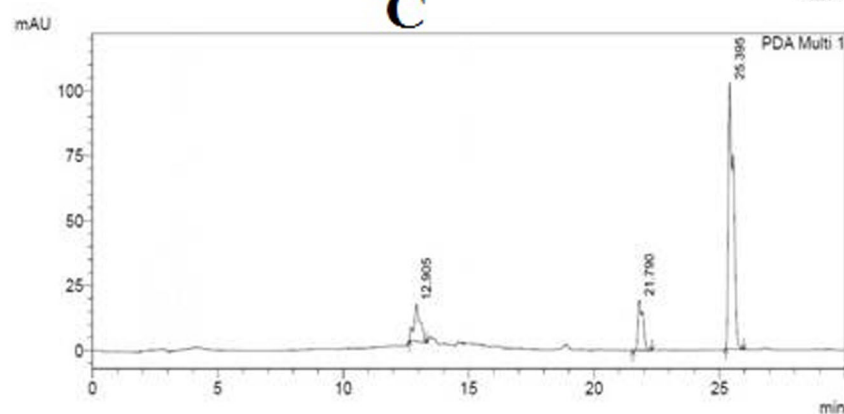**D**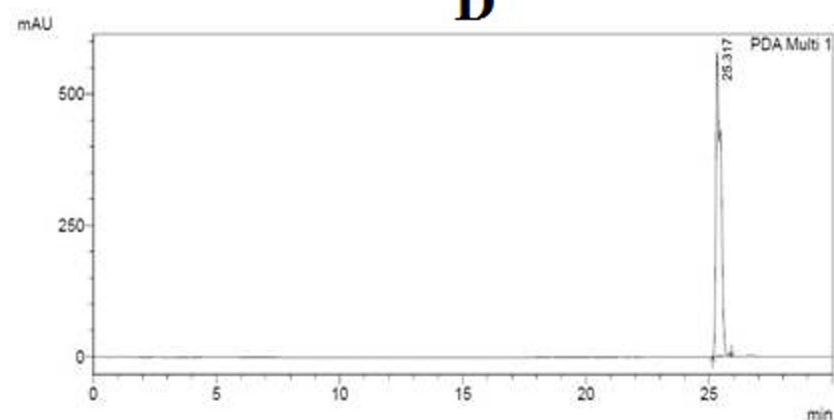**F**

| Extract/standard | Retention time |
|------------------|----------------|
| Chrysophanol     | 25.317         |
| ECT              | 25.395         |
| Emodin           | 21.799         |
| ECT              | 21.790         |
| Kaemferol        | 14.944         |
| ECT              | 14.964         |

**E**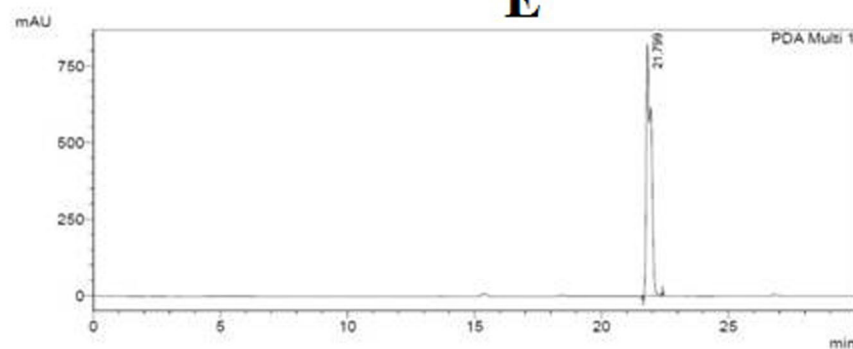

Supplement: Annie_Abraham_et_al_supplemental_content.zip [file IPHB_A_1299769_SM9214.zip › Annie Abraham et al supplemental content.pdf]
